# Immigration reduces selection in water microbial community assembly

**DOI:** 10.3389/fmicb.2024.1508136

**Published:** 2025-01-07

**Authors:** Fen-Guo Zhang, Kefan Wu, Sanqing Zhang, Furong Liang, Zhihua Du, Yongji Wang, Quan-Guo Zhang

**Affiliations:** ^1^College of Life Science, Shanxi Engineering Research Center of Microbial Application Technologies, Shanxi Normal University, Taiyuan, China; ^2^State Key Laboratory of Earth Surface Processes and Resource Ecology, MOE Key Laboratory for Biodiversity Science and Ecological Engineering, College of Life Sciences, Beijing Normal University, Beijing, China

**Keywords:** water bacteria, microcosm, immigration, environmental selection, dispersal limitation, dispersal swamping

## Abstract

To investigate the influence of immigration on the selection in structuring local water bacterial communities, we conducted a new community assembly experiment using microcosms filled with sterile original water medium under outdoor conditions. We collected air particulate matter from dust pooled from samples collected at 10 locations across ~20 km in a warm temperate region in Linfen City (northern China). The immigration rates were increased by introducing air particulate matter into the microcosms. The diversity, structure, and composition of the bacterial community in the water were assessed using 16S rRNA gene amplicon sequencing on the 13th and 60th days after the start of the experiment. Our results showed that increasing immigration did not lead to significant changes in the overall diversity of the total bacterial community on the 13th day. However, on the 60th day, diversity significantly increased. The variation explained by the environment substantially decreased from the 13th to the 60th day. The amount decreased from the control to the high immigration treatments, with a range of 65.0 to 29.8% on the 13th day and 34.0 to 15.4% on the 60th day. The dominant phyla differed significantly. In the early stage, Proteobacteria (69.6%) accounted for a higher relative average abundance, while Firmicutes (4.6%), Cyanobacteria (6.0%), Planctomycetota (8.1%), Verrucomicrobiota (2.0%), and Halobacterota (0.9%) were more abundant in the late stage. Additionally, the late stage had an average of 33 phyla, compared to 15 phyla in the early stage. All the results suggested a minimal role of dispersal limitation in structuring water bacterial communities in the early stage, whereas, in the late stage, the bacterial communities might experience dispersal swamping in our study area. Variance partitioning indicated that throughout the experiment, increasing immigration weakened the signal of environmental selection in the water microbial community assembly. These results expand our understanding of the impact of immigration on environmental selection and provide insights into the varying importance of dispersal and selection on microbial community assembly at different stages of succession.

## 1 Introduction

One of the most fundamental questions in microbial ecology is how microbial community diversity is generated and maintained. The traditional view in microbial ecology is that free-living microbes do not experience dispersal limitation; instead, niche processes determine the local community structure (de Wit and Bouvier, 2006; O’Malley, 2008; Fukami, 2015). This idea has been recently challenged by a substantial body of research (Yannarell and Triplett, 2005; Martiny et al., 2006; Nemergut et al., 2013). Studies on microbial biogeography have found that geographic distance and contemporary environmental dissimilarity often jointly explain the spatial variation in microbial community composition. They suggest that both dispersal limitation and niche processes have played important roles in the assembly of microbial communities. Specifically, an increase in dissimilarity in community composition with physical distance, when environmental differences were statistically controlled, was considered evidence for dispersal limitation (Papke et al., 2003; Whitaker et al., 2003; Yannarell and Triplett, 2005; Martiny et al., 2006). However, observational studies have limitations that may make their conclusions controversial. For example, it is difficult, if not impossible, to identify and measure all the ecologically relevant environmental factors, particularly biotic factors (Hanson et al., 2012; Zhao et al., 2019). Additionally, there may be a lack of independence between geographic distance and environmental dissimilarity (Martiny et al., 2006; Chase and Myers, 2011). As a result of these constraints, the majority of observational studies only assess the relative importance of environmental and geographical factors, leaving the role of stochastic processes in community assembly largely unresolved (Yannarell and Triplett, 2005; Langenheder and Ragnarsson, 2007).

An experimental approach is a powerful tool for testing the independent effects of a specific process. Our previous research tested whether the local bacterial communities were dispersal-limited using a propagule addition approach (Zhang et al., 2019). Some studies used microcosms with controlled aquatic medium exposed to atmospheric immigration with different exposure periods before further incubation to investigate the relative importance of stochastic immigration/dispersal limitation and environmental filtering (Bell, 2010; Lee et al., 2013; Székely et al., 2013). Langenheder and Székely used sterile natural aquatic medium from various sources and detected the signature of environmental filtering (Langenheder and Székely, 2011; Székely et al., 2013). Zhao found that local biotic interactions were responsible for species-specific divergence in soil bacterial communities through an abundance-manipulation experiment (Zhao et al., 2019). Nonetheless, these experimental studies were unable to provide a comprehensive understanding of microbial community assembly (Lindström and Langenheder, 2012). The challenge remains in determining whether a local community is dispersal-limited, and further understanding how immigration rates influence during assembly of water microbial communities (Zhou and Ning, 2017).

**Figure 1:**
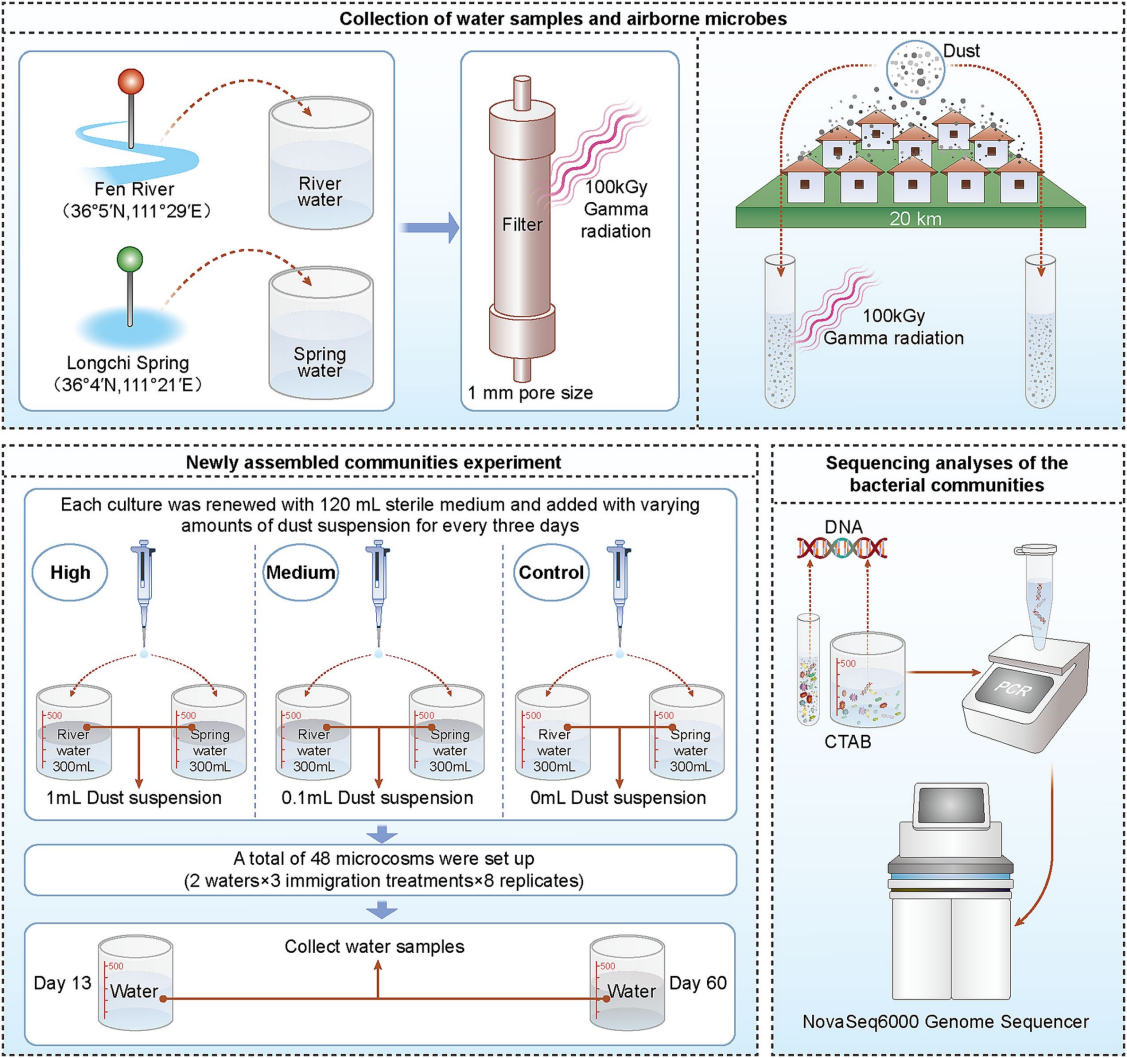
Visual representation of the experimental design.

The most direct method to test whether a local community has experienced dispersal limitation is through a regional species pool addition experiment. If the species diversity of the community increases after adding a regional species pool, it can be concluded that the community has experienced a restriction due to dispersal limitation (Hubbell and Borda-de-Água, 2001; Myers and Harms, 2009; Fukami, 2015). In this study, we collected air particulate matter, specifically dust samples, from 10 locations within a warm temperate region, spanning approximately 20 km (Myers and Harms, 2009; Choudoir et al., 2018; Zhang et al., 2019). The local microbe immigration rates were controlled by adding air particulate matter to the microcosms. We collected two water samples from a specific location and used sterile measuring glasses as microcosms to simulate a new community assembly experiment (Figure 1).

In evolution, some studies suggested that a moderate increase in gene flow can enhance genetic variation, which serves as the raw material for natural selection, and consequently facilitates local adaptation. However, excessive gene flow can disrupt natural selection and potentially result in local maladaptation, a phenomenon known as swamping by gene flow (Haldane, 1956; Lee-Yaw et al., 2016). Coincidentally, in metacommunities, the mass effect archetype focuses on how maladaptive (but perhaps unavoidable) exchanges of individuals among patches with different local conditions can enhance local diversity. This occurs when metacommunity diversity is high, by generating source-sink relations among local populations (Shmida and Wilson, 1985; Loreau and Mouquet, 1999). As shown in Supplementary Figure 1, when immigration is limited, obvious environmental selection may not be observed. In a particular habitat, perhaps only one maladaptive species has established a population without any other species immigrating. In this case, each species does not necessarily live in its most adaptable environment. When immigration is not limited, obvious environmental selection signals may be observed. When immigration rates are excessively high, it leads to an increase in the number of species inhabiting the environment, including some maladaptive species that are sustained by ongoing immigration. In this case, the impact of environmental selection is also weak (Loreau and Mouquet, 1999; Mouquet and Loreau, 2003). Based on the above theory in metacommunity and the community assembly experiment, our study aimed to test the following hypotheses: (1) in the early stage, the bacterial communities of local water are more likely to experience dispersal limitation (vs. weak selection); and thus (2) increasing immigration should enhance selection signal in the water microbial community assembly.

## 2 Materials and methods

### 2.1 Collection of water samples and airborne microbes

Water samples came from Fen River (36°5′N, 111°29′E) and Longci Spring (36°4′N, 111°21′E) in Linfen City (northern China) in June 2020. These samples were filtered through a 1-mm pore size filter and used to set up microcosms. The water samples were sterilized (by 100 kGy gamma radiation), stored at 4°C, and then used as a medium for culture renewal. The sterility of the water and dust mixture was confirmed by enumeration of culturable bacteria on nutrient agar plates (3 g L^−1^ of beef extract, 10 g L^−1^ of peptone, 5 g L^−1^ of NaCl, and 15 g L^−1^ of agar), as well as by extracting DNA and amplifying the V4 hypervariable region of the 16S rRNA gene. The checking results showed that the DNA was thoroughly destroyed after sterilizing by 100 kGy gamma irradiation (Zhang and Zhang, 2015, 2016; Zhang et al., 2019). The physical and chemical properties of sterilized water samples were detected (Supplementary Table S1). The community structure of bacteria in water samples was analyzed by sequencing.

Airborne microbes within 20 km of the experiment site were obtained by collecting outdoor dust on 10 buildings that had not been disturbed by humans for a long time in June 2020. These building sites cover an area of one degree of latitude and two degrees of longitude (Supplementary Table S2). Microbe sampling tools contained dual-tipped sterile swabs and a Ziplock bag. A homogenized mixture of the 10 dusts was created (with equal amounts of dust from every site), referred to as a “regional air pool” (Choudoir et al., 2018). The dust mixture was stored in centrifuge tubes at −20°C until processed. Parts of the mixture were sterilized by 100 kGy gamma radiation. The community structure of bacteria in dust was analyzed by sequencing.

Microcosm experiments were conducted outdoors at the Shanxi Normal University campus in July and August 2020. All microcosms were placed in a 3 m × 3 m area.

### 2.2 Experiment on newly assembled communities

Every initial microcosm contained sterile water from one sampling site. Sterilized river water and spring water were loaded into glass beakers (300 ml of water in 500 ml sterile measuring beakers). The microcosms were open to air (lids removed). Each culture was renewed with 120 ml of sterile medium every 3 days. One elevated immigration (high immigration) treatment was given to one-third of the microcosms: at each transfer, each microcosm received suspensions of dust of 1 ml. The second elevated immigration (medium immigration) treatment was given to another one-third of the microcosms: at each transfer, each microcosm received suspensions of the dust of 0.1 and 0.9 ml of sterilized suspensions. The ambient immigration (control) treatment microcosms received sterilized suspensions of dust of 1 ml.

A total of 48 microcosms were set up in this experiment, including 2 waters, 3 immigration treatments, and 8 replicates. Samples were collected on day 13 (transfer 4) and day 60 (transfer 20) for bacterial sequencing analysis of the community structure. The abbreviations for each group were as follows: FRS0 (local regional pool), FRS1 (control of Fen River environment on day 13), FRS2 (medium immigration of Fen River on day 13), FRS3 (high immigration of Fen River on day 13), FRS4 (control of Longci Spring environment on day 13), FRS5 (medium immigration of Longci Spring on day 13), FRS6 (high immigration of Longci Spring on day 13), LRS1 (control of Fen River environment on day 60), LRS2 (medium immigration of Fen River on day 60), LRS3 (high immigration of Fen River on day 60), LRS4 (control of Longci Spring environment on day 60), LRS5 (medium immigration of Longci Spring on day 60), and LRS6 (high immigration of Longci Spring on day 60; Supplementary Date 1, sheet1).

### 2.3 Sequencing analyses of the bacterial communities

Fen River and Longci Spring water samples, as well as dust and microcosm water samples, were used for total DNA extraction, with the method of CTAB. We amplified the V4 hypervariable region of the 16S rRNA gene with the universal primer pair 515F (5′-GTGCCAG CMGCCGCGGTAA-3′) and 806R (5′-GGACTACHVGGGTWTC TAAT-3′), tagged with unique barcodes for each sample (Caporaso et al., 2011). Polymerase chain reactions (PCRs) were conducted in a 30-μl reaction volume containing 15 μl (2×) of Phusion Master Mix, 3 μl (2 μM) of Primer, ~10 ng of template DNA, and 2 μl of ddH_2_O. The samples were initially denatured at 98°C for 1 min, amplified for 30 cycles of 10 s at 98°C, 30 s at 50°C, and 30 s at 72°C, followed by a final 5-min extension at 72°C. The PCR products were agarose gel verified and purified using the GeneJET DNA QIAquick Gel Extraction Kit (Thermo Fisher Scientific, Massachusetts, USA). For each sample, the two replicate DNA extracts were amplified and pooled into one sample for amplicon sequencing.

Sequencing was performed on an Illumina NovaSeq6000 Genome Sequencer (paired-end 250 bp; Novogene Technology Co., Ltd., Tianjin, China). Paired-end reads were assigned to the samples based on their unique barcode and truncated by cutting off the barcode and primer sequence. Paired-end reads were merged using FLASH (V1.2. 1 1; Magoc and Salzberg, 2011), a very fast and accurate analysis tool designed to merge paired-end reads when at least some of the reads overlap the read generated from the opposite end of the same DNA fragment, and the splicing sequences were called raw tags. Quality filtering on the raw tags was performed using the fastp (Version 0.23.1) software to obtain high-quality Clean Tags (Bokulich et al., 2012). The tags were compared to the reference database (Silva138.1) using the UCHIME Algorithm to detect chimera sequences, and then the chimera sequences were removed (Edgar et al., 2011). Then, the effective tags were finally obtained. For the effective tags, denoise was performed using the DADA2 module in the QIIME2 software (Version QIIME2-202202) to obtain initial amplicon sequence variants (ASVs), which were binned at a 100% similarity (Wang et al., 2001). Species annotation was performed using QIIME2 software with the Silva database (Silva 138.1), and the results were further corrected through the NCBI database (the download time was 2021.9). With the classify-sklearn algorithm in the QIIME2 software, each ASV was annotated using a pre-trained Naive Bayes classifier.

### 2.4 Data analysis

ASV richness, Pielou’s evenness, the Shannon index, and differences in community composition were estimated using rarefied ASV tables with a depth of 51,589 sequences per sample. These estimations were performed using the vegan package (Oksanen et al., 2008) in the R environment (R Core Team, 2014). The Bray–Curtis dissimilarity index (Bray and Curtis, 1957) was used to infer differences in bacteria taxa composition between water samples. The pairwise dissimilarity between sample i and j is calculated as BC_ij_ = 1 − 2C_ij_/(S_i_ + S_j_), where C_ij_ is the number of ASVs in common between sample i and j, and S_i_ and S_j_ are the total numbers of ASVs counted in sample i and j, respectively. To generate dissimilarity matrices, pairwise Bray–Curtis indices were calculated for 48 communities across three immigration treatments at two-time points.

The following statistical analyses were conducted to investigate the impact of immigration on environmental selection based on community dissimilarities at three different immigration treatments. The statistical analyses were performed using the vegan package (Oksanen et al., 2008) in R (R Core Team, 2014). Permutational multivariate analysis of variance (PERMANOVA) was used with the Adonis function to partition the variation in bacterial communities into variations explained by these two factors at two-time points (Anderson, 2001). The F statistic, which is analogous to Fisher’s F-ratio, was calculated using the community dissimilarity matrix. The *p*-value was obtained based on 9,999 permutations. Non-metric multidimensional scaling (NMDS) plots were derived to represent the dissimilarity (weighted UniFrac distance) between microcosms (Kruskal, 1964). The immigration, environment, and temporal information for each community provided an intuitive visual illustration of how these factors influenced community variation and how community dissimilarities changed over time.

A cluster tree was constructed using UPGMA, which was based on the weighted UniFrac distance matrix. This method is commonly used in ecology for evolutionary classification. The UPGMA diagram was generated using the upgma.tre function within QIIME.

## 3 Results

### 3.1 Microbial community alpha diversity

**Figure 2:**
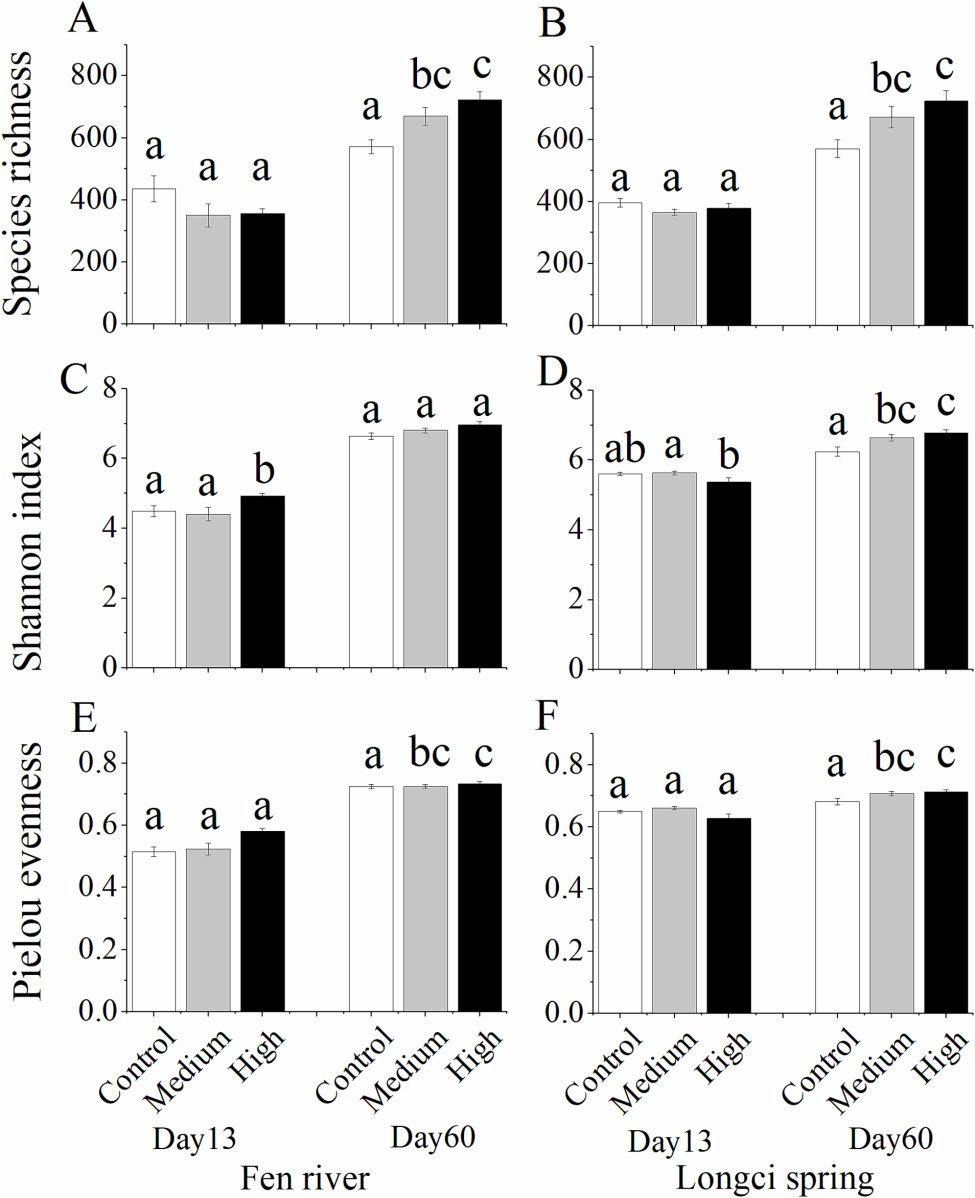
Species richness **(A–B)**, Pielou’s evenness **(C–D)**, and Shannon index **(E–F)** of bacterial communities in the experimental microcosms of control, medium, and high immigration treatments on the 13th and 60th days. **(A,C,E)**, the Fen River water microcosms. **(B, D,F)**, the Longci Spring water microcosms. Differences were estimated using one-way ANOVA, and means were compared using Tukey’s test. Data in **(A–F)** show mean ± SE (*n* = 8). In each panel, the same alphabet represents an insignificant difference between the two treatments.

Among the 48 microcosms, on the 13th day, the richness of ASVs did not change for either the Fen River (*F_2, 23_* = 2.064, *p* = 0.152) or Longci Spring microcosms according to three different immigration treatments (*F_2, 23_* = 1.365, *p* = 0.277, Figures 2A,B, day 13). Pielou’s evenness significantly changed for both the Fen River (*F_2, 23_* = 5.969, *p* = 0.009) and Longci Spring microcosms (*F_2, 23_* = 3.506, *p* = 0.049, Figures 2C,D, day 13). The Shannon index did not show any difference for either the Fen River (*F_2, 23_* = 3.459, *p* = 0.051) or Longci Spring microcosms (*F_2, 23_* = 2.950, *p* = 0.074, Figures 2E,F, day 13). On the 60th day, the richness of ASVs significantly increased for both the Fen River (*F_2, 23_* = 8.736, *p* = 0.002) and Longci Spring microcosms (*F_2, 23_* = 5.848, *p* = 0.010, Figures 2A,B, day 60). Pielou’s evenness did not show any difference for the Fen River microcosms (*F_2, 23_* = 0.579, *p* = 0.569), but significantly increased for the Longci Spring microcosms (*F_2, 23_* = 3.956, *p* = 0.035, Figures 2C,D, day 60). The Shannon index significantly increased for both the Fen River (*F_2, 23_* = 3.774, *p* = 0.040) and Longci Spring microcosms (*F_2, 23_* = 6.657, *p* = 0.006, Figures 2E,F, day 60). Overall, according to three immigration treatments, on the 13th day, the diversity of bacterial communities did not significantly differ, however, significantly increased on the 60th day. The results suggested that in the early stage, the bacterial communities of local water were not dispersal-limited, whereas they experienced dispersal swamping in the late stage (Figure 2). This was contrary to our assumption that the bacterial communities of local water are more likely to experience dispersal limitation in the early stage.

### 3.2 Beta diversity analysis of microbial communities

The community variation across environments was statistically analyzed using non-parametric multivariate analysis of variance (MANOVA; Table 1). The environment factor had significant effects on the community variation at all three immigration treatments (*p* ≤ 0.001), indicating the importance of environmental selection in community assembly. It is worth noting that from the 13th to the 60th day, the variation explained by the environment substantially decreased across all three immigration treatments. Additionally, the amount of variation explained by the environment decreased from the control to the high immigration treatments, with a range of 65.0 to 29.8% on the 13th day and 34.0 to 15.4% on the 60th day (Table 1). This suggests that immigration reduced environment selection, which was contrary to our assumption that increasing immigration should enhance the selection signals in the water microbial community assembly.

**Table 1:** Non-parametric MANOVA of the Bray–Curtis dissimilarities among experimental communities at each immigration rate.

		Environment						Residuals
		*Df*	*SS*	*MS*	*F*	*p*	*R^2^*	
Control	Day 13	1	1.127	1.127	25.996	0.001^***^	0.650	0.350
	Day 60	1	0.513	0.513	7.206	0.001^***^	0.340	0.660
Medium immigration	Day 13	1	0.850	0.850	19.035	0.001^***^	0.576	0.424
	Day 60	1	0.248	0.248	2.547	0.003^**^	0.154	0.846
High immigration	Day 13	1	0.468	0.468	5.929	0.001^***^	0.298	0.702
	Day 60	1	0.341	0.341	4.770	0.001^***^	0.254	0.746

*F*, *F*-test statistic; *p*, proportion of randomization trials with more extreme values of *F*; *R*^2^, explainable proportions by a certain factor. **A *p*-value of less than 0.01 indicates a highly significant difference. ***A *p*-value of less than 0.001 indicates an extremely significant difference.

The community variation across environments and immigration rates was statistically analyzed using MANOVA (Table 2). The environment had a noticeable effect on community composition (PERMANOVA tests that included both environment and immigration microcosms; day 13, *F_2, 47_* = 33.058, *p* = 0.0001; day 60, *F_2, 47_* = 9.060, *p* = 0.0001). Furthermore, the immigration rate caused a detectable change in community composition (day 13, *F_2, 47_* = 5.295, *p* = 0.0001; day 60, *F_2, 47_* = 6.167, *p* = 0.0001), with a similar effect observed for the interaction between environment and immigration rate (day 13, *F_2, 47_* = 5.439, *p* = 0.0001; day 60, *F_2, 47_* = 2.356, *p* = 0.0008). The percentage of variation in microbial communities explained by immigration and environment was 11.0 and 34.2% on the 13th day, and 18.1 and 13.3% on the 60th day, respectively. These results show that environmental selection played a larger role in compositional variation in the early stage, while immigration was more significant in the late stage (Table 2). This also indicates that immigration reduced environment selection, which was contrary to our assumption that increasing immigration should enhance the selection signal in water microbial community assembly.

**Table 2:** Non-parametric MANOVA of the Bray–Curtis dissimilarities among experimental communities within two factors.

Source of variance	Day 13						Day 60					
	*Df*	*SS*	*MS*	*F*	*R^2^*	*P*	*Df*	*SS*	*MS*	*F*	*R^2^*	*P*
Environment	1	1.839	1.839	33.058	0.342	0.0001^***^	1	0.725	0.725	9.060	0.133	0.0001^***^
Immigration	2	0.589	0.295	5.295	0.110	0.0001^***^	2	0.988	0.494	6.167	0.181	0.0001^***^
Environment: immigration	2	0.605	0.303	5.439	0.113	0.0001^***^	2	0.377	0.189	2.356	0.069	0.0008^***^
Residuals	42	2.337	0.056		0.435		42	3.363	0.080		0.617	
Total	47	5.370			1.000		47	5.453			1.000	

*Df*, degrees of freedom; *SS*, sums of squares; *MS*, mean squares; *F*, *F*-test statistic; *R*^2^, explainable proportions by a certain factor; *p*, proportion of randomization trials with more extreme values of *F*. ***A *p*-value of less than 0.001 indicates an extremely significant difference.

The variation of the community across different environments, immigration rates, and time was also statistically analyzed using MANOVA (Supplementary Table S3). All three factors resulted in noticeable changes in community composition (*p* < 0.001), as did their interactions (*p* < 0.01). Time, environment, immigration, and the interaction of time and environment accounted for 51.8, 3.8, 3.5, and 7.6% of the overall variation, respectively (Supplementary Table S3). The results suggested that immigration might influence environment selection signals and result in tremendous differences in the community structure between the early and late stages.

**Figure 3:**
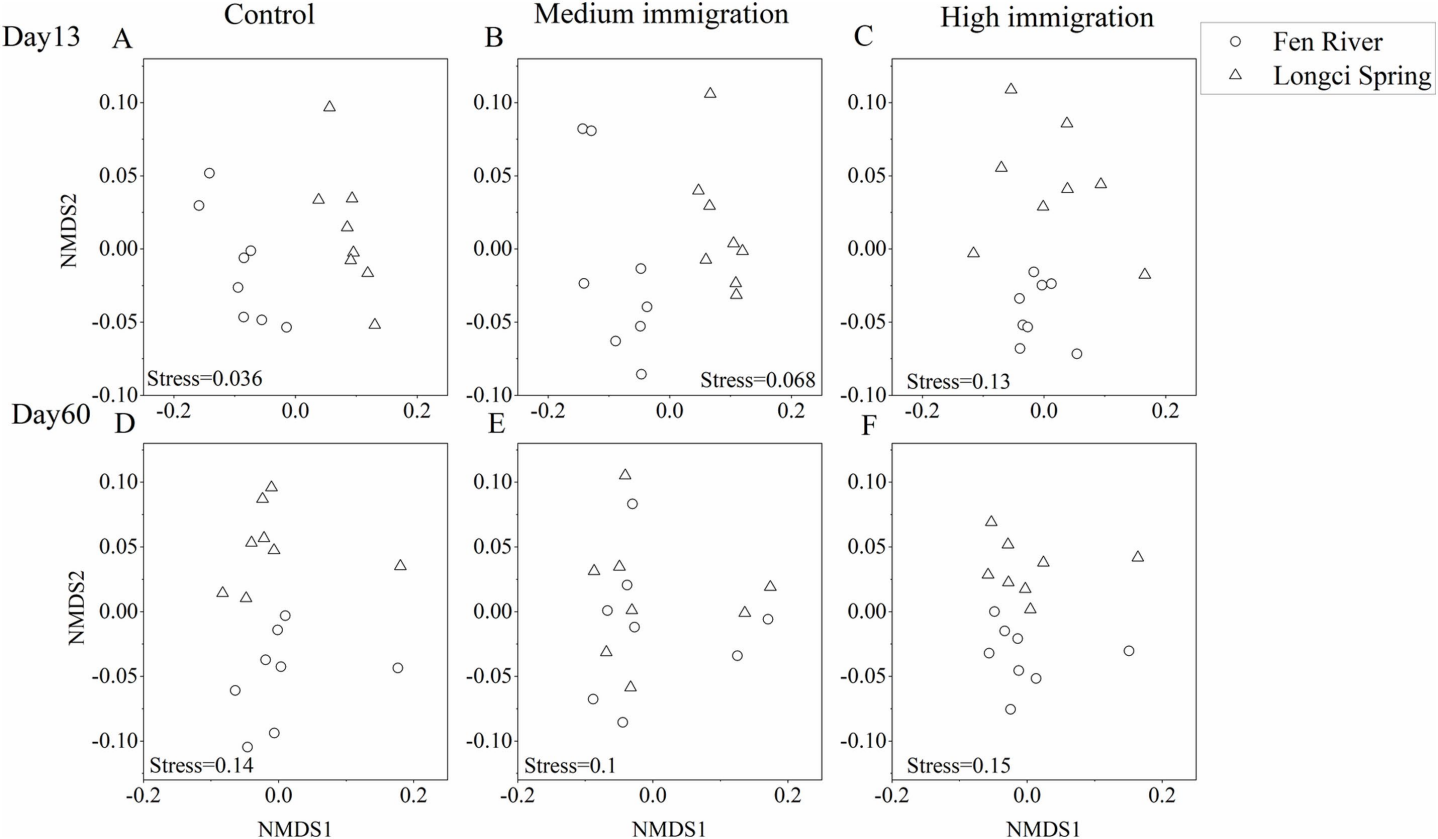
Non-metric multidimensional scaling ordination of experimental communities on the 13th **(A–C)** and the 60th **(D–F)** day for each immigration rate. Each point represents an experimental community, and the distance between two points represents the dissimilarities between communities. Stress is a function assessing how well the derived two-dimensional plot fits the pairwise dissimilarity matrix (stress >0.2, poor; stress = 0.1, fair; stress <0.05, good; stress = 0, perfect). The pairwise dissimilarities of all communities at a specific immigration rate are shown, and points having the same shape were communities from the same environment.

**Figure 4:**
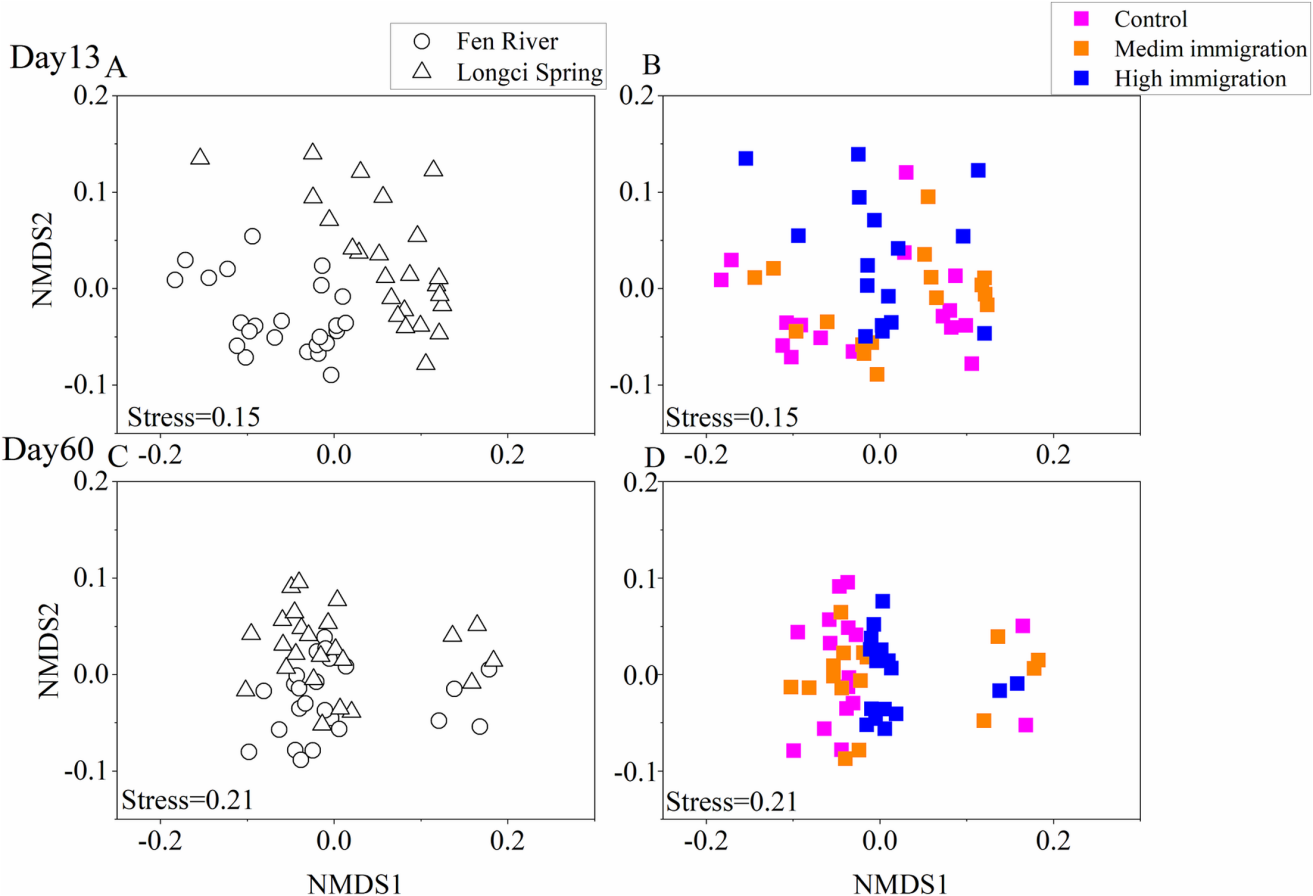
Non-metric multidimensional scaling ordination of experimental communities on the 13th **(A,B)** and the 60th **(C,D)** day for environment and immigration treatments. Each point represents an experimental community, and the distance between two points represents the dissimilarities between communities. Stress is a function assessing how well the derived two-dimensional plot fits the pairwise dissimilarity matrix (stress >0.2, poor; stress = 0.1, fair; stress<0.05, good; stress = 0, perfect). The pairwise dissimilarities of all communities at a specific environment or immigration rate are shown; in **(A,C)**, points having the same shape were communities from the same environment (hollow circle represented sterilized Fen River water medium and hollow triangle represented sterilized Longci Spring water medium), and in **(B,D)**, points having the same color were communities from the same immigration rate.

Non-metric multidimensional scaling (NMDS) was utilized to generate a visual representation of the dissimilarities between every pair of communities. It was observed that communities within the same environment and immigration rate tended to aggregate, further supporting the conclusion that environment significantly influenced community assembly across three immigration rates at two different time points (Figure 3). Moreover, when examining the dissimilarities between each pair of communities across various environments and immigration rates on the 13th and 60th days, it was evident that communities within the same environment but with different immigration rates exhibited a greater tendency to aggregate. Similarly, communities with the same immigration rate but different environments also displayed a higher likelihood of aggregating. This suggests that both environment and immigration played substantial roles in community assembly on the 13th and 60th days (Figure 4).

### 3.3 Microbial community composition

**Figure 5:**
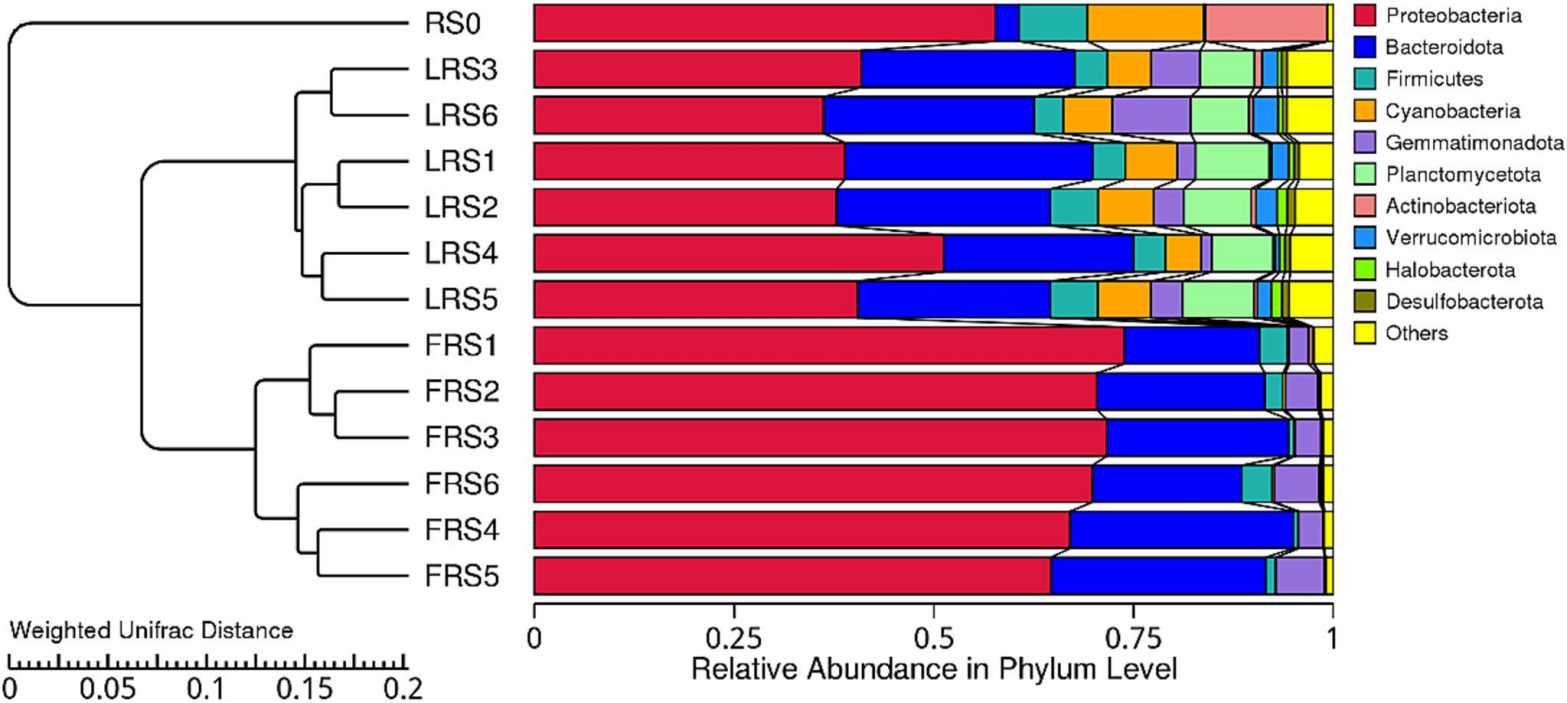
UPGMA clustering tree for each group at the phylum level. The weighted UniFrac distance matrix was used for UPGMA cluster analysis, and the cluster results were integrated with the relative species abundance of each group at the phylum level. The left side of the figure shows the structure of the UPGMA cluster tree, while the right side displays the distribution map of species relative abundance for each group at the phylum level.

The dominant phyla differed significantly among the regional air pool and the microcosms on the 13th and 60th days. In the early stage, Proteobacteria (69.6%) accounted for a higher relative average abundance, while Firmicutes (4.6%), Cyanobacteria (6.0%), Planctomycetota (8.1%), Verrucomicrobiota (2.0%), and Halobacterota (0.9%) were more abundant in the late stage. In the regional air pool, Firmicutes (8.6%), Cyanobacteria (14.5%), and Actinobacteriota (15.3%) were found to be the highest (Figure 5). Additionally, the late stage had an average of 33 phyla, compared to 15 phyla in the early stage (Supplementary Data 1, sheet3).

## 4 Discussion

### 4.1 No dispersal limitation in the early stage and dispersal swamping in the late stage

Among the 48 microcosms from Fen River and Longci Spring water environments, on the 13th day according to three immigration treatments, the ASVs richness and Shannon index did not significantly differ overall (Figure 2). The results suggested that, at the early stage, the bacterial communities in local water were not dispersal-limited. This was contrary to our assumption that in the early stage, the bacterial communities in local water would be more likely to experience dispersal limitation. However, it aligned with our previous study, which suggested a negligible role of dispersal limitation in structuring soil bacterial communities (Zhang et al., 2019). This might be because natural microorganism immigration was already quite high (Mathew and Jonathan, 2018), and these species were likely to have inherently high dispersal rates in the early stage. On the 60th day, the ASV richness, Pielou’s evenness, and the Shannon index significantly increased overall (Figure 2). The results demonstrated that in the late-stage excessive immigration resulted in a higher diversity of bacterial communities, indicating a phenomenon known as “dispersal swamping” (Figure 2).

### 4.2 Immigration reducing selection

The results of MANOVA suggested that the environmental selection signal was less noticeable in the late stage compared to the early stage, which was contrary to our assumption that increasing immigration should enhance the selection signal in the water microbial community assembly (Tables 1, 2; Figures 3, 4). The results could be explained by considering the mass effects in the metacommunity (Supplementary Figure S1). At the early stage, the natural air microorganism immigration was sufficient at the control treatment, so that the bacterial communities in local water didn’t experience dispersal limitations and strong environmental selection signal was observed. Dispersal enabled a locally inferior species to persist in an environment with a superior competitor as long as there was spatial variation in competitive abilities resulting from abiotic heterogeneities or biotic variance (e.g., priority effects). Although local population growth should be negative, the presence of microbial immigration did not significantly affect the diversity of the three immigration treatments. In the late stage, continuous immigration rates were too high, leading to an increase in the number of species in the environment, including some maladaptive species sustained by ongoing immigration. Consequently, diversity significantly increased with increasing immigration. However, due to dispersal swamping, the influence of environmental selection was weaker in the late stage compared to the early stage. If our experiment had been conducted for longer than 60 days and immigration had continued to increase, competition outcomes may have been reversed, with inferior competitors from the metacommunity overpowering locally superior competitors through their high dispersal rates. This phenomenon, known as swamping, could result in an overall reduction in local and regional diversity, as species with more efficient dispersal abilities outcompete locally more competitive species simply due to their sheer numbers (Mouquet and Loreau, 2003).

Our MANOVA results indicated that increasing immigration (from control to the high) consistently reduced the influence of environment selection. In the early stage, selection accounted for more of the compositional variation than immigration, whereas the opposite was true in the late stage (Tables 1, 2; Figures 3, 4). Changes in community composition were detectable due to the effects of environment, immigration rates, time, and their interactions (Supplementary Table S3). Thus, our results demonstrated that immigration might reduce environment selection signals and result in a tremendous difference in community structure between the early and late stages. This was contrary to our assumption that increasing immigration should enhance the selection signal in the water microbial community assembly. The reason might be that natural microorganism immigration was already quite high in the early stage, and these pioneer species suitable for growth were likely to have inherently high dispersal rates. Therefore, increasing immigration would interfere with environmental selection. As species, especially autotrophic bacteria and algae, established populations, more species were able to thrive. The pioneer species altered the abiotic environment, allowing a variety of species in the “secondary succession” stage to inhabit any type of habitat, thus reducing the signal of abiotic environmental selection. Even in the late stage, increasing immigration still had the effect of diminishing the signal of environmental selection.

The fact that our experimental communities within the same environment still showed substantial compositional variation (Tables 1, 2; Supplementary Table S3) highlights the important role of ecological drift effects. Additionally, the variation explained by the environment decreased significantly with the immigration rate increasing from the 13th to the 60th day (Table 1), which essentially emphasized the drift process in our experiment. Furthermore, there was substantial compositional variation among the replicated communities from the same environment and immigration rate, suggesting that drift played a central role in structuring the communities (Supplementary Figure S2). It is important to note, however, that a large amount of the unexplained variation does not necessarily indicate the importance of the drift process in some observational studies (Langenheder and Ragnarsson, 2007; Bell, 2010; Caruso et al., 2011; Jia et al., 2020), as there may be several unmeasured environmental and/or historical parameters at play. Nonetheless, in our experimental system, we controlled for environmental heterogeneity by using two types of water (Supplementary Table S1). Given that atmospheric factors, such as illumination and temperature, remained consistent throughout our experimental system, no environmental factors were left unmeasured. Additionally, the experimental microcosms were effectively isolated, with no exchange of medium between them, making unmeasured spatial factors unlikely. Therefore, a large portion of the variation that was unaccounted for by the immigration rate, environment, and time in our experiment can be confidently interpreted as drift processes (Hao et al., 2016).

Our results also imply that immigration, selection, and drift interacted directly or indirectly and worked together to shape community structure in our experiment. The strength of selection depended on the other two processes. Environmental selection and drift varied in opposite directions, with drift being more important when selection was weak. The importance of drift in shaping community structure also relied on immigration rates, with drift playing a larger role when the immigration rate was high, leading to an increased chance of stochastic variations among local communities (Tables 1, 2). Dispersal limitation, which was absent in our experiment, could potentially overshadow the influences of environmental selection on community structure and not create community variation on its own without being coupled with the drift process (Tables 1, 2). In our study, immigration reduced selection, and the reducing role might be influenced by drift as well. Our findings are supported by numerous studies (Chase and Myers, 2011; Hanson et al., 2012; Nemergut et al., 2013; Stegen et al., 2013; Graham et al., 2016; Evans et al., 2017; Menéndez-Serra et al., 2023).

For the 10 most abundant phyla, the relative abundance varied greatly among the regional air pools and microcosms on the 13th and 60th days. In the early stage, microcosms within the same environment exhibited close aggregation, while in the later stage, microcosms with the high immigration treatment showed close aggregation (Figure 5). The relative abundance of Proteobacteria was higher in the early stage, whereas Firmicutes, Cyanobacteria, Planctomycetota, Verrucomicrobiota, and Halobacterota were higher in the late stage. Firmicutes, Cyanobacteria, and Actinobacteriota were most abundant in the regional air pool (Figure 5). Additionally, the average number of phyla in the late stage was higher than that in the early stage (Supplementary Data 1, sheet 3). These results suggest that differences in community composition may explain why environment, immigration rates, and time affected the water microbial community assembly, with increased immigration weakening the signal of environmental selection.

## 5 Conclusion

Our results suggested a negligible role of dispersal limitation in structuring water bacterial communities in the early stage, whereas, in the late stage, the bacterial communities in our study area might be susceptible to dispersal swamping. Thus, increasing immigration weakened the environmental selection signal in the assembly of water microbial communities throughout the entire experiment. These findings contribute to our understanding of the processes underlying variations in community composition and diversity, emphasize the significance of immigration in selection, and offer insights into the concept of mass effects in metacommunity theory. Future research should aim to investigate all processes involved in the assembly of eukaryotic microbial communities and gain a comprehensive understanding of the community structure of all microorganisms (Vass et al., 2020; Groult et al., 2022).

## Data Availability

The datasets presented in this study can be found in online repositories. The names of the repository/repositories and accession number(s) can be found in the article/Supplementary material.
