# Supplementary material for: Immigration reduces selection in water microbial community assembly
Source: Front Microbiol. 2025 Jan 7;15:1508136. doi: 10.3389/fmicb.2024.1508136 (PMC11747843; doi:10.3389/fmicb.2024.1508136)
Supplement: Supplementary file 1 [file Presentation_1.pdf]

Supplementary Material

Fen-Guo Zhang[1], Kefan Wu[1], Sanqing Zhang[1], Furong Liang[1], Zhihua Du[1], Yongji Wang[1*], Quan-Guo Zhang [2*]

[1]College of Life Science, Shanxi Engineering Research Center of Microbial application technologies, Shanxi Normal University, Taiyuan, Shanxi, China

[2]State Key Laboratory of Earth Surface Processes and Resource Ecology and MOE Key Laboratory for Biodiversity Science and Ecological Engineering, College of Life Sciences, Beijing Normal University, Beijing, China

*** Correspondence:**

Yongji Wang: yongjisxnu@126.com, Quan-Guo Zhang: zhangqg@bnu.edu.cn

Supplementary Data included in separate files:

1 Supplementary Data

Supplementary Data 1

This file includes:

2 Supplementary Figures and Tables

2.1 Supplementary Figures

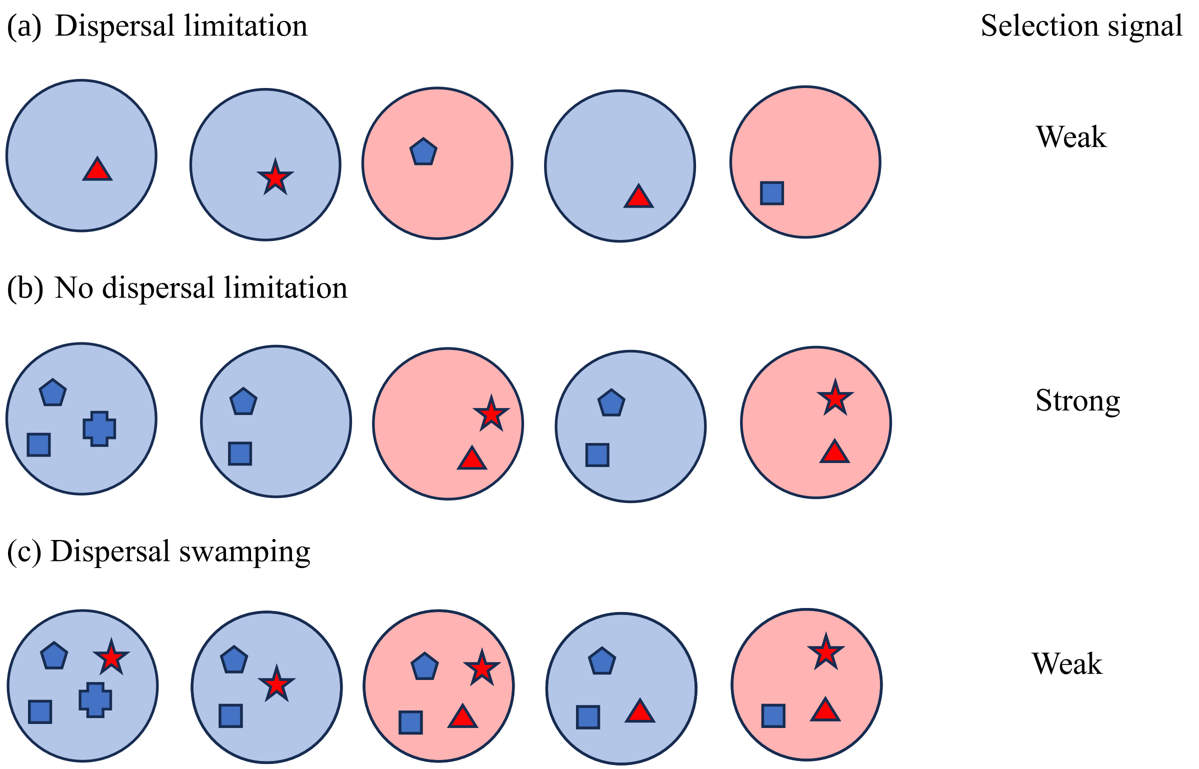

Supplementary Figure 1. The relationship between dispersal and selection signal. (a) Dispersal limitation vs weak selection signal, (b) No dispersal limitation vs strong selection signal, and (c) Dispersal swamping vs weak selection signal. Big light blue and red circles represent two distinct habitats. Small circles of various colors and shapes represent different species. Blue species are better suited for living in the light blue habitat, while red species are more adapted to the light red habitat. The selection outcomes are indicated by the matching between the species and habitats.

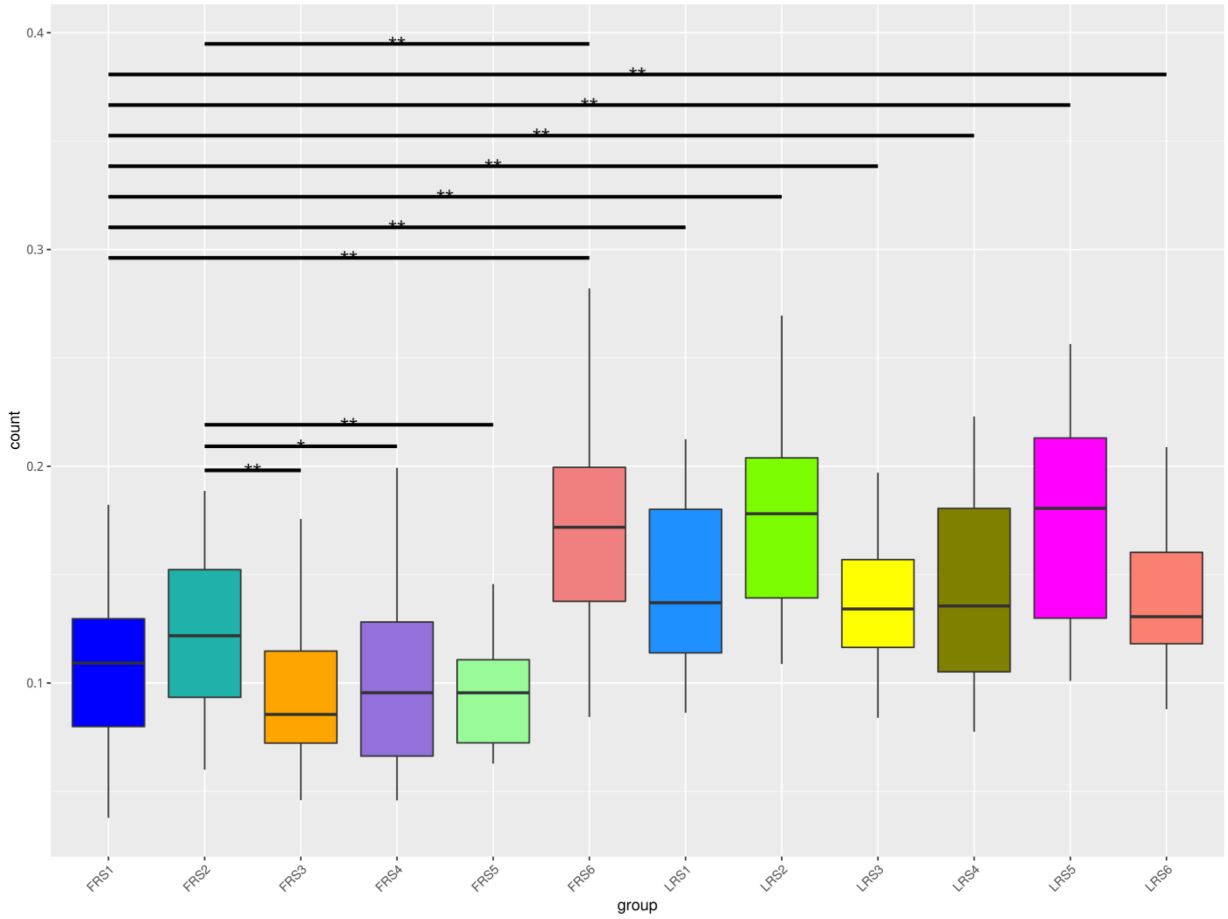

Supplementary Figure 2. Beta-diversity based on weighted unifrac distance for every group. Significant differences between groups were shown by kruskalWallis test. * *p* <0.05, ** *p*<0.01, *** *p* <0.001.

2.2 Supplementary Tables

Supplementary Table 1. Physical and chemical properties of water samples

Index	Medium	
	Longci Spring water	Fen River water
DO	12.6	11.0
AN	0.040	1.050
COD_{Cr}	4	15
TP	0.18	0.16
TN	2.67	2.48
pH	7.34	7.22
T	20.0	29.0

Note: DO: Dissolved oxygen (mg/L); AN: Ammonia nitrogen (mg/L); COD_{cr}: Chemical oxygen demand (mg/L); TP: Total phosphorus (mg/L); TN: Total nitrogen (mg/L); T: Temperature (℃)

Supplementary Table 2. Information about the 10 sites to collect dust for the regional microbial pool

Site ID	Location	Altitude (m)
A	111°05′ N; 35°56′ E	449
B	111°29′ N; 36°06′ E	449
C	111°29′ N; 36°01′ E	447
D	111°10′ N; 36°10′ E	445
E	111°33′ N; 36°31′ E	448
F	111°20′ N; 36°04′ E	450
G	111°47′ N; 36°20′ E	449
H	111°29′ N; 36°04′ E	447
I	111°43′ N; 35°55′ E	440
J	111°15′ N; 35°58′ E	450

Supplementary Table 3. Non-parametric MANOVA analysis of the Bray–Curtis dissimilarities among experimental communities within three factors.

**Source of Variance**	***Df***	***SS***	***MS***	***F***	R^2	***P***
Environment	1	0.849	0.849	12.511	0.038	0.0001***
Immigration	2	0.785	0.392	5.781	0.035	0.0001***
Time	1	11.624	11.624	171.325	0.518	0.0001***
Environment:Immigration	2	0.415	0.208	3.058	0.018	0.0084**
Environment:Time	1	1.716	1.716	25.286	0.076	0.0001***
Immigration:Time	2	0.792	0.396	5.837	0.035	0.0002***
Environment:Immigration:Time	2	0.567	0.284	4.181	0.025	0.0015**
Residuals	84	5.699	0.068		0.254	
Total	95	22.447			1.000	

Abbreviations: *Df*, degrees of freedom; *SS*, sums of squares; *MS*, mean squares; *F*, F-test statistic; R^2, explainable proportions by certain factor; *P*, proportion of randomization trials with more extreme values of F.
